# Giant Benign Phyllodes Tumor Presenting with Skin Ulceration: A Case Report and Review of the Literature

**DOI:** 10.70352/scrj.cr.26-0307

**Published:** 2026-07-15

**Authors:** Minori Kawabata, Kenichiro Tanaka, Shiori Tohyama, Ryunosuke Akima, Riku Yamamoto, Mamiko Miyashita, Hisaki Kato, Shunsuke Sakuraba, Ryo Wada, Tomoaki Ito, Goro Kutomi

**Affiliations:** 1 Department of Surgery, Juntendo University Shizuoka Hospital, School of Medicine, Juntendo University, Izunokuni, Shizuoka, Japan; 2 Department of Pathology, Juntendo University Shizuoka Hospital, School of Medicine, Juntendo University, Izunokuni, Shizuoka, Japan; 3 Department of Breast Oncology, Juntendo University Hospital, School of Medicine, Juntendo University, Tokyo, Japan

**Keywords:** phyllodes tumor, giant breast tumor, benign breast tumor, skin ulcer

## Abstract

**INTRODUCTION:**

Phyllodes tumors of the breast are rare fibroepithelial neoplasms accounting for 0.3%–1.0% of all primary breast tumors. They are classified into benign, borderline, and malignant subtypes, with borderline and malignant tumors associated with an increased risk of local recurrence and distant metastasis. Giant phyllodes tumors, defined as tumors larger than 10 cm in diameter, are uncommon, and reports of tumors exceeding 20 cm are extremely rare. We report a case of a rapidly growing giant benign phyllodes tumor with skin ulceration, along with a review of the relevant literature.

**CASE PRESENTATION:**

A 48-year-old Asian woman presented with rapid enlargement of the left breast and active bleeding from an ulcerated mass. Clinical findings and imaging studies revealed a well-circumscribed giant tumor measuring 24 cm that occupied the entire left breast, without evidence of distant metastasis. Core needle biopsy suggested a fibroepithelial tumor, and a benign phyllodes tumor was most strongly considered. A left mastectomy was performed. Skin grafting had been considered preoperatively, as the skin defect would be large. However, as sufficient skin flaps were obtained intraoperatively, due to the skin being stretched by the tumor, only mastectomy was done without a reconstructive procedure. Histopathological examination confirmed a benign phyllodes tumor with negative surgical margins. The postoperative course was uneventful.

**CONCLUSIONS:**

We experienced a rare case of a giant breast tumor and tumor resection. Although rapid breast tumor growth and skin breakdown suggested malignancy, the pathological diagnosis was a benign phyllodes tumor. This highlights the importance of considering phyllodes tumors in the differential diagnosis of rapidly enlarging breast masses and the need for timely surgical intervention. This case is particularly noteworthy because it demonstrates an unusual combination of clinical features—giant tumor size, rapid growth, and skin ulceration—that are generally associated with malignant disease, despite the final diagnosis being benign.

## Abbreviations

AJCC
: American Joint Committee on Cancer
WLE
: wide local excision

## INTRODUCTION

Phyllodes tumors of the breast are fibroepithelial neoplasms characterized by the proliferation of both ductal epithelial and stromal components. They account for approximately 0.3%–1.0% of all primary breast tumors.^1)^ Phyllodes tumors are classified into benign, borderline, and malignant subtypes, with borderline and malignant tumors showing a higher possibility for local recurrence and distant metastasis.^2,3)^ “Giant phyllodes tumors” are defined as lesions exceeding 10 cm in diameter and, according to previous reports, comprise 20% of all phyllodes tumors.^1,2)^

We encountered an exceptionally large breast tumor, 24 cm in diameter, accompanied by skin ulceration and hemorrhagic discharge from the exposed lesion. To our knowledge, only a few cases of phyllodes tumors exceeding 20 cm in diameter have been reported. Herein, we report a case of a rapidly growing giant benign phyllodes tumor with skin ulcerations treated at our institution, a local university hospital, along with a review of the relevant literature on giant phyllodes tumors.

## CASE PRESENTATION

A 48-year-old Asian woman was referred from another local hospital nearby with progressive enlargement of the left breast accompanied by active bleeding from the ulcerated part of the tumor. The patient had been transported to the previous hospital 1 month earlier because of rapid breast enlargement and continuous bleeding from the tumor. While initial evaluation was being performed at the previous hospital, the mass was noted to have enlarged, though the precise timeline and growth rate could not be independently verified at our institution. Surgical management with skin grafting was considered. As the previous hospital did not have a plastic surgery department, she was referred to our hospital, which has a plastic surgery department capable of performing reconstructive procedures. The patient had a history of epilepsy and mild developmental disability and lived at home under the care of her parents. Her performance status was PS 0–1, and she was physically capable of independent activity. No motor paralysis was present. Despite her preserved physical function, her developmental disability and epilepsy may have limited her awareness of the tumor’s progression and her ability to seek medical attention independently, potentially contributing to a prolonged untreated period during which the tumor grew to its remarkable size. She had no family history of breast or ovarian cancer.

Physical examination revealed an enlarged left breast tumor 24 cm in diameter, comparable in size to a volleyball, accompanied by a foul odor (**Fig. 1A**). The tumor extended inferiorly toward the abdomen, and the overlying skin was erythematous, with partial protrusion of the lesion through the skin surface (**Fig. 1B**). Hemorrhagic discharge from the ulcerated skin was also observed. The right breast appeared normal.

**Fig. 1:**
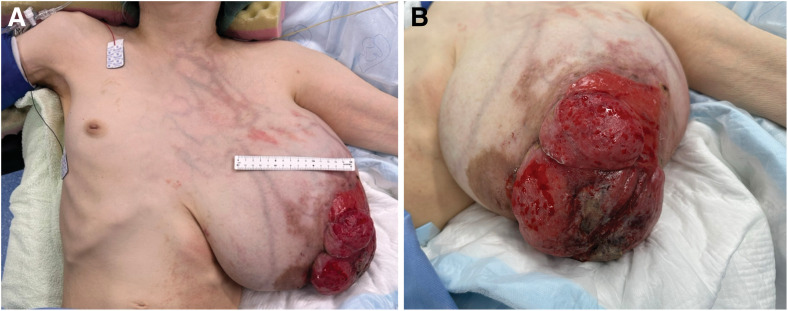
Initial physical examination. (**A**) Physical examination revealed an enlarged left breast 24 cm in diameter, comparable in size to a volleyball, accompanied by a foul odor. (**B**) The tumor also extended inferiorly toward the abdomen, and the overlying skin was erythematous, with partial protrusion of the lesion through the skin surface. Hemorrhagic discharge from the ulcerated skin was also observed.

Mammography was not performed due to the massive size of the tumor. Ultrasound performed at our hospital revealed a well-circumscribed, multi-lobular shaped mass measuring 23 × 21 cm, almost occupying the entire left breast. The tumor showed heterogeneous enhancement with significant vascular proliferation (**Fig. 2**). No significant lymph node enlargement was observed on either side. These ultrasonographic findings suggested a phyllodes tumor; however, malignancy could not be excluded at this point. No specific features were observed in the right breast.

**Fig. 2:**
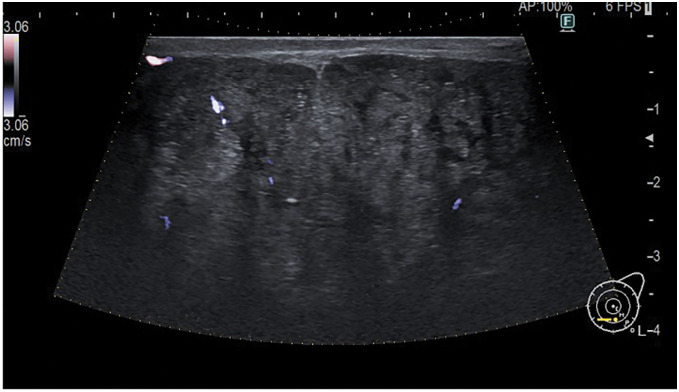
Sonography of the left breast. Ultrasound performed at our hospital revealed a well-circumscribed, multi-lobular shaped mass measuring 23 × 21 cm, occupying the entire left breast. The internal echogenicity of the tumor was heterogeneous, with marked internal vascularity.

Contrast-enhanced MRI of the mammary gland revealed a giant tumorous lesion in the left breast, demonstrating heterogeneous enhancement with an internal septum and a characteristic “leaf-like” contrast enhancement pattern. These findings suggested the diagnosis of a phyllodes tumor. It was difficult to clearly identify the fat plane between the lesion and the chest wall musculature because of the little intervening fat. However, there were no obvious irregular margins suggestive of direct tumor invasion into the chest muscle layer. The subcutaneous fat of the left breast was partially preserved, although it was extremely thin or absent in areas surrounding the ulcerated region (**Fig. 3**).

**Fig. 3:**
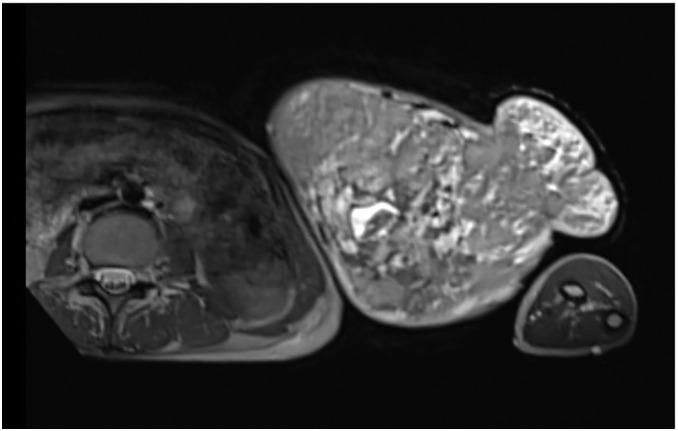
Contrast-enhanced MRI of the mammary gland (axial T2-weighted image). Contrast-enhanced MRI of the mammary gland revealed a giant tumorous lesion in the left breast, demonstrating heterogeneous enhancement with an internal septum and a characteristic “leaf-like” contrast enhancement pattern. There were no obvious irregular margins suggestive of direct tumor invasion into the chest muscle layer. The subcutaneous fat of the left breast was partially preserved, although it was extremely thin or absent in areas surrounding the ulcerated region.

Additionally, contrast-enhanced CT showed a tumorous lesion exceeding 20 cm in maximum diameter in the left breast. The internal density was heterogeneous, and partial cystic components were observed. The lesion was in broad contact with the pectoral muscle, and therefore muscle invasion could not be excluded. Multiple nodules were observed in the left axilla, the largest measuring approximately 8 mm in short-axis diameter. No clearly enlarged lymph nodes were identified in the supraclavicular fossa region. In addition, no evidence of distant metastasis was observed (**Fig. 4**). Taken together, the rapid tumor growth and relatively well-defined margins despite the large size suggested the most likely diagnosis to be a phyllodes tumor.

**Fig. 4:**
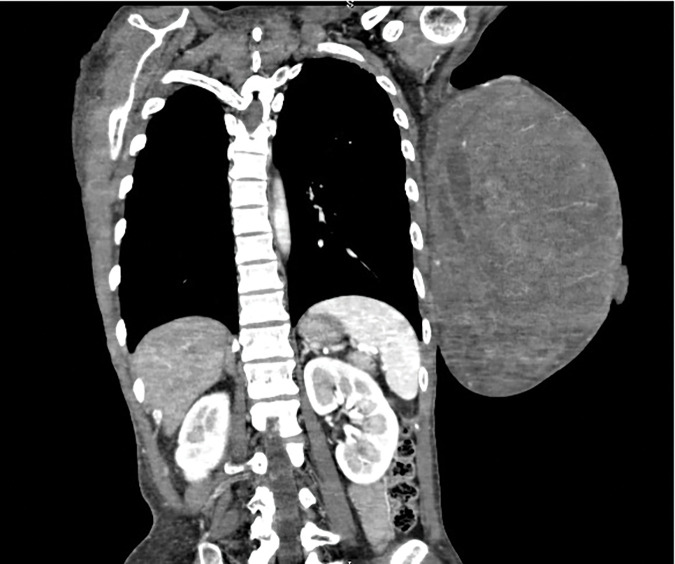
Axial view of contrast-enhanced CT of the chest. Contrast-enhanced CT demonstrated a giant heterogeneous mass exceeding 20 cm in the left breast with partial cystic components. The tumor was in broad contact with the pectoral muscle, and muscle invasion could not be definitively excluded. Small left axillary lymph nodes were noted, with the largest measuring approximately 8 mm in short-axis diameter, while no supraclavicular lymphadenopathy or distant metastasis was identified.

Core needle biopsy of the left breast lesion was performed at the previous hospital. The biopsy was performed targeting the peripheral portion of the tumor under ultrasound guidance, as the central area was suspected to contain necrotic or cystic components less suitable for histological assessment. Reevaluation of the specimen at our hospital showed scattered glandular structures with edematous fibrous stroma in the background. No clusters of markedly atypical cells were identified. The overall findings suggested fibroadenoma or benign phyllodes tumor.

Given the large tumor size occupying the entire breast, total mastectomy was planned from the outset rather than lumpectomy, as complete tumor removal with adequate margins would not have been achievable with breast-conserving surgery. Regarding the extent of resection, as preoperative imaging did not demonstrate clear invasion of the pectoralis major muscle or direct skin infiltration, combined resection of either structure appeared to be unnecessary; however, given the remarkable tumor size, the possibility of requiring skin grafting was anticipated preoperatively, and plastic surgery was consulted accordingly.

A left mastectomy was performed through a Stewart transverse skin incision. Intraoperatively, the tumor was found to occupy the entire breast with almost no residual normal breast tissue, and no macroscopic adhesion to the pectoralis major muscle or infiltration of the overlying skin, allowing complete preservation of both structures. Although skin grafting was considered preoperatively, adequate skin flaps were obtained intraoperatively, due to the skin being stretched by the tumor, and the tumor was easily dissectible from the pectoralis fascia with no adhesion or invasion; therefore, the procedure was completed with mastectomy alone. The excised specimen measured 25.0 × 26.0 × 16.0 cm and weighed 4.95 kg (**Fig. 5A** and **5B**). **Figure 5A** and **5B** display the specimen with orientation markers indicating the superior, inferior, medial, nipple area, and lateral margins. Macroscopic observation of the cross-sectional surface demonstrated a well-demarcated tumor border throughout all margins, with no evidence of chest wall invasion. These macroscopic findings were subsequently confirmed by postoperative pathological examination, which revealed no invasive components at the surgical margins or infiltration into the surrounding breast parenchyma or pectoralis fascia. The postoperative course was uneventful, and the patient was discharged on POD 7.

**Fig. 5:**
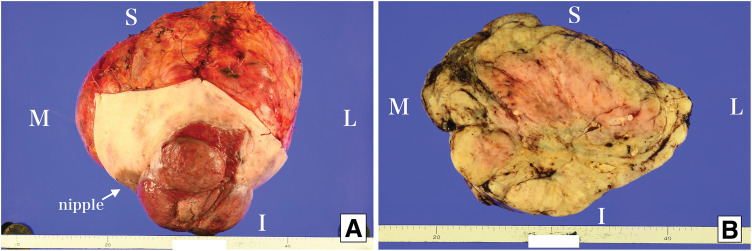
Macroscopic findings of the resected specimen (25.0 × 26.0 × 16.0 cm, 4.95 kg). **(A)** Macroscopic image of the excised specimen with orientation markers indicating the superior (S), inferior (I), medial (M), and lateral (L) margins and nipple area. The specimen demonstrates well-demarcated borders throughout. **(B)** Cross-sectional view of the specimen demonstrating a well-demarcated tumor border throughout all margins, with no evidence of infiltration into the surrounding breast parenchyma or pectoralis fascia, consistent with the absence of invasive components confirmed on postoperative pathological examination.

Final histological examination was as follows. The lesion measured 24.0 cm in maximum dimension with negative surgical margins (**Fig. 6A** and **6B**). The mass consisted of fibroepithelial components with focal “leaf-like” structures. Although no prominent cytological atypia was identified, additional immunohistochemical studies were performed using p40, Ki-67, and p53. The proliferative glandular tissue showed preserved biphasic differentiation with myoepithelial cells (**Fig. 6C**). Immunohistochemically, p40 negativity confirmed the absence of squamous differentiation, a low Ki-67 labeling index indicated low proliferative activity, and p53 negativity was inconsistent with malignant transformation (**Fig. 6D** and **6E**). Overall, these findings were consistent with a diagnosis of benign phyllodes tumor.

**Fig. 6:**
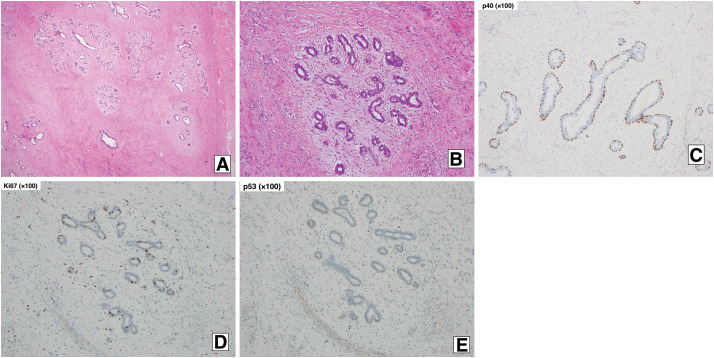
Histopathological examination. The mass consisted of fibroepithelial components with focal leaf-like structures. Although no prominent cytological atypia was identified. (**A**) H & E stain (×40), (**B**) H & E stain (×100), and (**C**) basal cell marker p40 (×100). The proliferative glandular tissue showed preserved biphasic differentiation with myoepithelial cells. (**D**) Ki-67 (×100). The Ki-67 labeling index was low. (**E**) p53 (×100). No clustered population of p53-positive cells were identified. H & E, hematoxylin and eosin

## DISCUSSION

Our case was a rare giant phyllodes tumor, larger than 10 cm in diameter. It was preoperatively suspected as a malignant lesion from clinical findings, because of its rapid growth and ulceration of the skin. Intraoperatively, skin grafting was planned; however, as the skin was extended due to the tumor growth, the procedure turned out to be unnecessary. Postoperative pathologic findings confirmed the diagnosis of benign phyllodes tumor.

Phyllodes tumors are a rare type of fibroepithelial tumor, accounting for less than 1% of all breast tumors.^1,2)^ They were first described by Johannes Müller as “cystosarcoma phyllodes” in 1838.^4–6)^ Histological features include proliferation of both epithelial and stromal components and characteristic “leaf-like” papillary projections; hence, it is called “phyllodes” tumor.^2,3,6)^ Common clinical presentation includes painless nodules and may also involve rapid growth. They are classified into benign, borderline, and malignant phyllodes tumors based on the assessment of the following 5 histological features: the degree of stromal cellular atypia, the mitotic activity per 10 high-power fields, infiltrative or circumscribed tumor margins, the presence or absence of stromal overgrowth, and the nature of the tumor borders.^5)^ It has been reported that no established criteria exist regarding the relationship between tumor size and the distinction between benign and malignant phyllodes tumors.^5)^ In our case, histopathological evaluation revealed mild stromal cellular atypia, without evident nuclear pleomorphism or marked cytological atypia. The mitotic activity was low, indicating no increased proliferative activity. The tumor demonstrated well-circumscribed, pushing margins, with no evidence of infiltrative growth into the surrounding breast parenchyma. Stromal overgrowth was absent, as no areas of stroma lacking epithelial components were identified. Overall, the tumor borders were smooth and relatively uniform, lacking irregular or infiltrative features, leading to a diagnosis of a benign phyllodes tumor. Ki-67 immunohistochemical staining, which reflects the proliferative activity of stromal cells, is considered to correlate well with the degree of malignancy of phyllodes tumors. The reported mean labeling indices are 3.6%–4.7% for benign phyllodes tumors, 13.9%–16% for borderline tumors, and 24.7%–50% for malignant tumors.^6)^ In our case, Ki-67 immunohistochemical staining was low, indicating the tumor to be benign.

The apparent discrepancy between the low Ki-67 labeling index and the rapid clinical tumor growth warrants discussion. Two possible explanations are proposed. First, the rapid macroscopic enlargement observed in the present case may be attributable to intratumoral infarct necrosis and hemorrhage rather than to intrinsic tumor cell proliferation. Infarct necrosis and hemorrhage in breast tumors, accompanied by edema, can arise from various mechanisms, including vascular occlusion, thrombosis, vasculitis, needle biopsy, relative ischemia, and tumor-specific immunological responses. Notably, benign breast tumors are more susceptible to infarction than malignant tumors, as the comparatively limited angiogenesis in benign lesions increases the risk of ischemic necrosis.^7)^ Thus, intratumoral necrosis, hemorrhage, and edema, rather than active cellular proliferation, may have driven the rapid increase in tumor volume observed clinically. Second, the large tumor size may reflect a prolonged untreated period of uncertain duration, during which gradual growth accumulated without medical intervention, rather than truly rapid proliferation. These 2 mechanisms are not mutually exclusive and may have acted in concert in the present case.

The majority of phyllodes tumors are benign, accounting for up to 75% of phyllodes tumors. Borderline and malignant phyllodes tumors account for up to 15%–20% and 10%–20% of all phyllodes tumors, respectively. The local recurrence rate for benign phyllodes tumors is 10%–20%,^8)^ while borderline and malignant phyllodes tumors are known to have higher rates of local recurrence (15%–40%) and metastasis (9%–27%).^9)^ It remains unclear whether histological features such as histological grade and the degree of stromal overgrowth are predictive markers for the frequency of local recurrence.^8)^

Initial diagnosis of phyllodes tumor is generally made by core needle biopsy; however, distinguishing phyllodes tumors from other fibroepithelial tumors, such as fibroadenomas, remains challenging.^1)^ Common fibroadenomas, which often stop growing at a certain size, can generally be managed with careful observation.^10)^ On the other hand, phyllodes tumors carry a risk of rapid growth and local recurrence and therefore often require surgical excision. Due to the differences in treatment approach, accurate preoperative differentiation between these tumors is clinically important.^8,10)^ In the present case, preoperative histopathological findings suggested the possibility of a phyllodes tumor, and surgical treatment was therefore indicated.

“Giant phyllodes tumors” are defined as tumors with a diameter larger than 10 cm and account for approximately 20% of all phyllodes tumors.^1,2)^ Although tumor size is often discussed as a potential indicator of malignancy, no definitive size threshold for malignancy has been established.^2,5)^

Several case reports have described giant benign phyllodes tumors measuring 20 cm or more in maximum dimension over the past 10 years (**Table 1**).^1,2,5,11)^ Sbeih et al. reported a 41-year-old patient with a giant benign phyllodes tumor measuring 20 × 20 × 25 cm, complicated by skin ulceration and severe breast disfigurement, which required mastectomy with skin grafting.^2)^ Fernández-Ferreira et al. reported a benign phyllodes tumor measuring 40.2 × 36.3 × 15.0 cm and weighing 4857 g, which to our knowledge represents 1 of the largest benign phyllodes tumors reported to date.^1)^ Fukuyama et al. described a case requiring emergency mastectomy for a giant benign phyllodes tumor weighing 2100 g, highlighting the potential for these tumors to occasionally necessitate urgent surgical intervention despite their benign histology.^5)^ Matei et al. reported a giant benign phyllodes tumor measuring 30 cm in a 24-year-old patient, treated with mastectomy and level I axillary lymph node sampling.^11)^ Collectively, these reports confirm that, although extremely rare, benign phyllodes tumors can reach enormous dimensions, and the present case—with a tumor measuring 25.0 × 26.0 × 16.0 cm and weighing 4950 g—is consistent with this body of literature.

**Table 1:** Reported cases of giant benign phyllodes tumors of the breast (≥20 cm)

No.	Author (year)	Age (years)	Tumor size (cm)	Tumor weight (g)	Surgical procedure	Skin grafting	Outcome during follow-up
1	Sbeih et al. (2015)^2)^	41	20 × 20 × 25	—	Mastectomy	Yes	—
2	Fukuyama et al. (2016)^5)^	63	30	2100	Emergency mastectomy	—	No recurrence
3	Fernández-Ferreira et al. (2021)^1)^	40	40.2 × 36.3 × 15.0	4857	Mastectomy	No	No recurrence
4	Matei et al. (2021)^11)^	24	30	—	Mastectomy with level I axillary lymph node sampling	No	—
5	Present case	48	25.0 × 26.0 × 16.0	4950	Total mastectomy	No	No recurrence

As demonstrated in the present case, some tumors may exhibit rapid growth and even skin ulceration while remaining histologically benign. In primary breast cancer, skin ulceration is generally considered a consequence of direct dermal invasion by the tumor, as reflected in its classification as T4b disease in the AJCC staging system. In contrast, benign breast tumors are characterized by expansile growth with well-circumscribed margins and do not demonstrate dermal or stromal invasion. Therefore, in giant benign breast tumors such as our present case, ulceration is thought to result from rapid tumor expansion exceeding the skin’s capacity to stretch. Progressive enlargement leads to increased tension, compromised perfusion, and eventually ischemic necrosis of the overlying skin, resulting in ulcer formation.^5)^ This mechanism likely explains why the skin defect was limited, as the skin was not extensively infiltrated by the giant tumor, but was simply “torn” by compression, which was 1 of the reasons skin grafting was not required in our case.

The standard treatment for phyllodes tumors is surgical excision.^1,2,5)^ Inadequate surgical margins have been considered as 1 of the most common risk factors for local recurrence.^1,2)^ Securing a negative surgical margin of approximately 1 cm, an approach known as WLE, is highly recommended to reduce the risk of local recurrence.^1,2,8,12)^ Mastectomy should be considered for giant phyllodes tumors when adequate margins cannot be achieved with breast-conserving surgery.^2)^ Careful postoperative follow-up is required even in cases with histologically negative surgical margins, as the possibility of local recurrence remains. Additionally, better outcomes with the preservation of the breast tissues and the nipple–areola complex could be expected with more timely diagnosis and treatment.^13)^

Despite being a benign phyllodes tumor, this case exhibited a large tumor size. This can be considered paradoxical compared to breast cancer, where tumor size generally correlates with malignancy.^14)^ Although there are no criteria explaining the correlation between tumor size and malignancy of phyllodes tumors,^5)^ this may be related to a prolonged period of illness. For example, other literature reports a 3-year interval between tumor awareness and consultation.^1)^ While the duration of illness in this case is unknown, it is likely prolonged given the patient’s reclusive lifestyle.

If a tumor does not invade surrounding tissues or metastasize like breast cancer, systemic deterioration is less likely to occur, potentially leading to cases where patients present only after local tumor enlargement.^14)^

However, the above explanation only partially accounts for the circumstances of this case, and further research findings are awaited.

## CONCLUSIONS

We encountered a rare case of a giant benign phyllodes tumor exceeding 20 cm in diameter with associated skin ulceration. Despite rapid tumor enlargement and skin breakdown, the final pathological diagnosis was benign. This case highlights the importance of considering phyllodes tumors in the differential diagnosis of rapidly enlarging breast masses and emphasizes the need for prompt surgical intervention. This case also suggests that tumor size does not necessarily correlate with malignancy in phyllodes tumors, unlike in breast cancer. However, to thoroughly explain this phenomenon, results of future studies are awaited.
